# Trustability for Resilient Internet of Things Services on 5G Multiple Access Edge Cloud Computing

**DOI:** 10.3390/s22249905

**Published:** 2022-12-16

**Authors:** Suleyman Uslu, Davinder Kaur, Mimoza Durresi, Arjan Durresi

**Affiliations:** 1 Department of Computer & Information Science, Indiana University Purdue University Indianapolis, Indianapolis, IN 46202, USA; 2 Department of IT, Mathematics and Statistics, European University of Tirana, 1000 Tirana, Albania

**Keywords:** trustworthy and resilient systems, trust management, internet of things sensor security, 5G multiple access edge computing security, attack mitigation

## Abstract

Billions of Internet of Things (IoT) devices and sensors are expected to be supported by fifth-generation (5G) wireless cellular networks. This highly connected structure is predicted to attract different and unseen types of attacks on devices, sensors, and networks that require advanced mitigation strategies and the active monitoring of the system components. Therefore, a paradigm shift is needed, from traditional prevention and detection approaches toward resilience. This study proposes a trust-based defense framework to ensure resilient IoT services on 5G multi-access edge computing (MEC) systems. This defense framework is based on the trustability metric, which is an extension of the concept of reliability and measures how much a system can be trusted to keep a given level of performance under a specific successful attack vector. Furthermore, trustability is used as a trade-off with system cost to measure the net utility of the system. Systems using multiple sensors with different levels of redundancy were tested, and the framework was shown to measure the trustability of the entire system. Furthermore, different types of attacks were simulated on an edge cloud with multiple nodes, and the trustability was compared to the capabilities of dynamic node addition for the redundancy and removal of untrusted nodes. Finally, the defense framework measured the net utility of the service, comparing the two types of edge clouds with and without the node deactivation capability. Overall, the proposed defense framework based on trustability ensures a satisfactory level of resilience for IoT on 5G MEC systems, which serves as a trade-off with an accepted cost of redundant resources under various attacks.

## 1. Introduction

**Figure 1:**
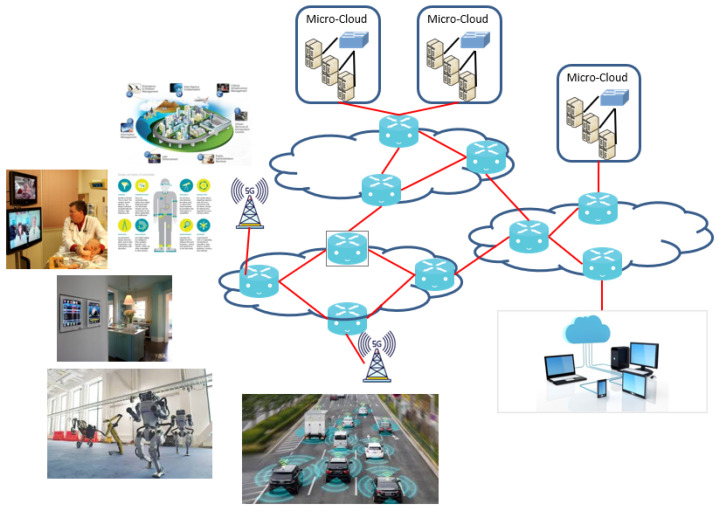
Internet of Things on 5G multiple-access edge computing systems.

Future fifth-generation (5G) wireless cellular networks will support billions of Internet of Things (IoT) sensors and devices, including static and mobile endpoints, various robots, and self-driving cars, as illustrated in Figure 1. These devices and the related applications will attract and amplify the risk of vulnerability. 5G wireless communication technologies under development promise tremendous improvements in many areas including speed, connectivity, and reduced latency. These 5G networks can enable the movement of massive amounts of data to connect distant sensors across a critical environment, as illustrated in Figure 1. IoT on 5G will function as an integrated system with multi-access edge computing (MEC) as an extension of cloud services. IoT and MEC systems will both be under various attacks; therefore, we propose a defense framework to ensure the resilience of the entire system under attack.

A variety of customers, such as individuals employing 5G devices for personal use or large corporations and institutions, are widely using cloud computing platforms. Therefore, a wide range of applications is migrated into the cloud, including e-commerce, data storage, healthcare, gaming, and different web applications [1,2]. This allows customers to deploy and scale their services with much less effort, especially without hardware purchase requirements [3]. However, it creates new concerns, such as with security and privacy [4,5]. These are the issues that customers consider before using cloud vendors and selecting the most appropriate provider. Therefore, as cloud providers recognize these issues, they develop their services such that they would address the customers’ concerns in order to attract them [1].

Similarly, devices and sensors that are connected via the internet or other types of connections are being widely adopted. The speed of this adaptation process is estimated to continue increasing as the new-generation networks such as 5G spread [6,7]. These 5G networks have had many advances in wireless networking [8,9,10,11].

There are a variety of sensors in such devices, whether it be an autonomous vehicle, a car with an active safety system, a robotic vacuum, or some other IoT device [12]. For such systems, the security of the communication between the decision makers and the sensors is critical. An attack on one of the sensors could cause undesirable outcomes specific to the task [13] or simply unauthorized access to sensitive information such as healthcare data [14].

Vendors have been implementing security measures in both cloud and systems with communicating parts [5]; however, it is difficult to completely protect the whole system from attackers [15]. Faced with such new challenges, the old security model of defending the system’s perimeter is no longer valid. We must assume that whatever defense mechanisms we deploy in the system will sooner or later be breached by attackers. Therefore, it is advisable to implement new techniques, including trustworthiness assessments [16], which would help the service to survive the attacks despite having to face the cost of these techniques, such as active tracking, dynamic resource allocation, and purchase of new resources. According to the Cybersecurity and Infrastructure Security Agency (CISA), the current cyberspace shifts the attention from detection and perimeter defenses to strengthening security with resilience [17,18]. A robust mechanism that ensures resilience is the deployment of redundant resources based on the assessment of the trustworthiness of the system services. Current methods may not adequately assess the trustworthiness of the systems and their components due to disproportionate heterogeneity and multi-level hierarchies.

Various studies [19,20,21] and surveys [22,23] have been carried out on trust management frameworks and their applications. Ruan et al. [24] proposed a measurement theory-based trust management framework to provide improved flexibility to context-dependent applications by supporting multiple formulations and a new metric: the confidence of trust. Applications of this framework include stock market prediction using Twitter data [25], trust management in environmental decision making [26,27,28], and the detection of crime [29], fake users [30], and damaging users [31]. These applications show the potential of utilizing a trust management framework to facilitate decision making in various fields by measuring and assessing trust.

Trust frameworks have also been proposed to be implemented in both cloud and IoT scenarios and other network-related ones, such as the scenarios that include 5G [32,33]. Ruan et al. also proposed a trust management framework for IoT [34], multi-access edge computing [35], and cloud computing platforms [36]. Furthermore, Kaur et al. [37] proposed the use of a geo-location and trust-based framework to filter out attackers in 5G social networks. These applications serve as stepping stones toward trustworthy artificial intelligence (AI) and decision making, which have been consistently promoted by researchers [38,39,40,41,42,43,44], governments, institutions, and organizations such as the European Union [45] and the International Organization for Standardization [46].

Park et al. [13] highlighted the significance of the security and privacy of communication and connectivity functions and proposed machine learning approaches to detect anomalies in in-vehicle networks. Furthermore, Cao et al. [47] surveyed the emerging threats in deep learning-based autonomous driving and listed different types of attacks on sensors, such as jamming and spoofing. In addition to 5G, research has been carried out and a framework has been proposed for sixth-generation (6G) networks, specifically investigating the technology’s applicability and the privacy concerns in relation to unmanned aerial vehicles (UAV) [48,49,50]. Ullo et al. [12] also highlighted the importance of intelligent environment monitoring systems that use IoT and sensors; however, vendors and providers would need precise metrics to take the necessary actions on time in order to assess the trustworthiness of the systems.

To address the concerns about measuring the different aspects of trustworthiness, the metrics of acceptance [51] and fairness [52] were proposed to facilitate environmental decision making, an explainability metric [53] was proposed to interpret AI medical diagnosis systems, and a trustability metric [54] to assess trust in cloud computing. This paper presents an extended version of the trust management framework that includes the trustability metric, which helps to take action when an external attack or an internal event occurs in an autonomous device equipped with sensors or in a service running on the cloud. First, a sampling subsystem is explained as part of an autonomous system consisting of one decision maker and two sensors; an attack on one of the sensors is simulated, and the change in the trustability of the sensor and the entire system is shown. Then, the simulation is repeated with increased redundancy by adding another sensor. Finally, another scenario is simulated, where the extra sensor can be activated later, for instance, when the sensor lifetime is essential.

The findings illustrate the utilization of the trust management framework and the trustability metric in multiple incident scenarios within a sample cloud structure. The sample cloud consisted of three nodes, where the trust of one of the nodes declined relatively, continually, or sharply for both a short and extended period of time. It was shown that the trustability metric captures the decline in trust in the entire service. Then, additional scenarios were explored, where extra nodes could be added to each task in order to keep the service trustability high with an increased expense. Furthermore, results were shown for the cloud that had the option to remove nodes, specifically the ones with low trust. Finally, the net utility metric was illustrated to compare these two scenarios with and without the node removal option. The main contributions of this paper are as follows.

- The trustability metric was demonstrated using a sampling subsystem with a sensor activation option, where an external attack occurs on a sensor;
- Different possible outcomes of internal incident scenarios were presented in a sample cloud environment, where the trustability of the service is tracked by the framework for each scenario;
- The trustability metric captured the trustability of the service whenever the cloud architecture allowed for the addition and removal of extra nodes for each task;
- The net utility function captured the need for additional nodes and helped to decide when to remove nodes in order to optimize the utility of the service;
- Overall, this paper proposes the use of a trust management framework with a trustability metric and a net utility function on a variety of external and internal incident scenarios in order to help take timely actions to keep the service alive and optimize the utility.

The rest of the paper is organized as follows. In Section 2, the trust management framework is introduced, which is tailored to measure the trust of sensors and nodes to capture overall trustability. In Section 3, the results of utilizing the framework and how it captures trustability are presented and discussed in (i) a subsystem with sensors, where an external attack occurs to a sensor, and (ii) a sample cloud, where internal incidents happen to a node. In Section 4, findings and contributions are summarized, and future work is discussed.

## 2. Materials and Methods

This section presents the trust management framework and how it is adapted to capture the trustability of a system or service while considering its cost and utility.

### 2.1. Trust Management Framework

In [24], a measurement theory-based trust management framework was proposed for online social communities. This framework has since been proven to facilitate decision-making in multiple areas such as online social networks [25], the food-energy-water nexus [44], crime detection [29], and cancer diagnosis [53]. It is a very flexible yet robust framework that can be adapted to different scenarios to capture trust.

The framework has two main components: impression, represented by *m*, and confidence, represented by *c*. The impression is the level of trust one party shows the other, and confidence is the degree of certainty of the impression. Although different formulations are possible [24], we selected the intuitive ones, as shown in Equations (1) and (2), in order to focus on the framework. In these equations, $m^{A:B}$, $c^{A:B}$, and $r_{i}^{A:B}$ represent the impression, confidence, and a measurement from *A* to *B*.

(1) $$\begin{array}{ccc} m^{A:B} & = & \frac{\sum_{i}^{N}r_{i}^{A:B}}{N} \end{array}$$

(2) $$\begin{array}{ccc} c^{A:B} & = & 1-2\sqrt{\frac{\sum_{i}^{N}{\left(m^{A:B}-r_{i}^{A:B}\right)}^{2}}{N(N-1)}} \end{array}$$

Trust measurements are context-dependent, which means that measurement needs to be precisely defined and specific to the context. In this study, the alternative ways of obtaining measurements [35] were combined, and predefined trust measurements were used to reflect the incidents better and to be able to concentrate on the framework and decisions. Furthermore, measurements were always normalized to be in [0–1], which caused the impression to remain in the same interval of [0–1] as an arbitrary unit.

### 2.2. Trust Management in Systems and Cloud

The proposed trust management framework can be adapted to scenarios where trust is assessed for parts of an autonomous system or nodes of a cloud. There are several proposed approaches [54] to measure trust, such as by measuring node flows, dividing them into incoming and outgoing, assigning different weights to such flows, and considering the trust of tasks inside of a node. Equation (3) shows that the trust of a node, $m_{node}$, can be measured as the weighted average of trust of the flow, $m_{flow}$, and tasks running on it, $m_{task}$. As shown in Equation (4), just as the trust of the tasks running on a node can affect its trust, the node itself can affect the trust in those tasks.

(3) $$\begin{array}{ccc} m_{node} & = & \frac{w_{flow}m_{flow}+\sum_{i}w_{task_{i}}m_{task_{i}}}{w_{flow}+\sum_{i}w_{task_{i}}} \end{array}$$

(4) $$\begin{array}{ccc} m_{task} & = & \frac{w_{flow}m_{flow}+\sum_{i}w_{node_{i}}m_{node_{i}}}{w_{flow}+\sum_{i}w_{node_{i}}} \end{array}$$

**Figure 2:**
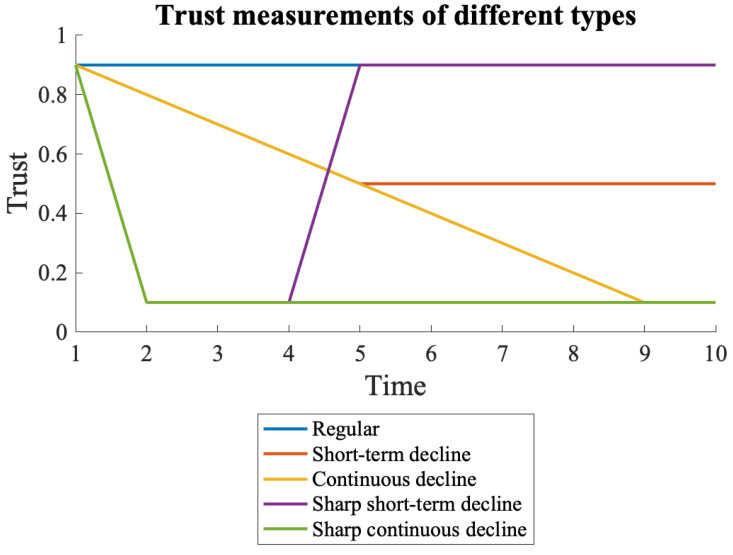
Trust measurements while different types of attacks occur.

In this study, trust measurements were simulated for different scenarios, such as a normal condition where the trust stays the same, with the exact high measurements of around 0.9 for 10 time intervals where time has an arbitrary unit for the absence of complication. Then, scenarios where the node received lower measurements were explored, reflecting an anomaly in either the flow or the task activity, which has two types: a short-term and a continuous decline in trust. Subsequently, additional scenarios where the node received very low measurements were explored. Equation (5) represents the examples of such trust measurements, which are also shown in Figure 2.

(5) $$\begin{array}{ccc} \mathrm{Regular} & = & \left\{0.9,0.9,0.9,0.9,0.9,0.9,0.9,0.9,0.9,0.9\right\}\\ \mathrm{Short-term}\;\mathrm{decline} & = & \left\{0.9,0.8,0.7,0.6,0.5,0.5,0.5,0.5,0.5,0.5\right\}\\ \mathrm{Continuous}\;\mathrm{decline} & = & \left\{0.9,0.8,0.7,0.6,0.5,0.4,0.3,0.2,0.1,0.1\right\}\\ \mathrm{Sharp}\;\mathrm{short-term}\;\mathrm{decline} & = & \left\{0.9,0.1,0.1,0.1,0.9,0.9,0.9,0.9,0.9,0.9\right\}\\ \mathrm{Sharp}\;\mathrm{continuous}\;\mathrm{decline} & = & \left\{0.9,0.1,0.1,0.1,0.1,0.1,0.1,0.1,0.1,0.1\right\} \end{array}$$

### 2.3. Redundancy, Cost, and Utility

The trust of the entire system was also explored, whether it be an autonomous system already deployed in the field or a cloud system that could be managed later on. The overall *trustability* of the system was measured by considering the individual trust of the nodes and their hierarchy, such that the nodes that were connected in series in logical representation were all required to have high trust, whereas the nodes connected in parallel compensated for each other’s abnormalities.

As discussed in [35], individual trustability was calculated using an exponential formula with two different lambdas, $\lambda_{1}$ and $\lambda_{2}$. This is because impression, *m*, and confidence, *c*, needed to be merged in order to reach a high trustability only when *m* and *c* were both high. Moreover, a threshold, ϕ, was provided for trustability to be adjusted for the application. In this study, the sample threshold used for demonstration was 0.5. In other words, the scenarios where *m* went below 0.5 had much lower trustability by using $\lambda_{2}$, whereas $\lambda_{1}$ was used otherwise. First, *m* and *c* were normalized and merged, as shown in Equation (6), where ϕ is the threshold. Then, trustability, τ, was calculated using the formula given in Equation (7), with the appropriate λ, which were assigned as $\lambda_{1}=4$ and $\lambda_{2}=8$. Trustability calculation is also shown in Algorithm 1.

(6) $$\nu =\frac{2(m-\phi )c+1}{2}$$

(7) $$\tau =e^{-\lambda (1-\nu )}$$

**Algorithm 1:** Trustability, τ, is calculated as an exponential function, where λ is decided by comparing the impression, *m*, with the threshold, ϕ. 
**Input**: m, c
 
**Output**: τ 
$\lambda_{1}\leftarrow 4$ 
$\lambda_{2}\leftarrow 8$ 
ϕ←0.5 
$v\leftarrow \frac{2(m-\phi )c+1}{2}$ 
**if** m≥ϕ **then**   |  
(
(
$\lambda \leftarrow \lambda_{1}$ 
**end** 
**else**   |  
$\lambda \leftarrow \lambda_{2}$ 
**end** 
$\tau \leftarrow e^{-\lambda (1-v)}$

After calculating individual trustability, the entire system’s trustability was calculated. First, the trustability of the nodes that were connected in parallel was aggregated, as shown in Equation (8). Then, the transitive trustability was calculated using the formula given in Equation (9). The final value reflects the entire system’s trustability, whether an active cloud system or an autonomous system that had been deployed in the field.

(8) $$\tau_{aggr}=1-\Pi_{i}\left(1-\tau_{i}\right)$$

(9) $$\tau_{tran}=\Pi_{i}\tau_{aggr_{i}}$$

Moreover, a formula to calculate the net utility of the service, including the cost of resources and the probabilities of success and failure, was developed using the trustability of the service. As shown in Equation (10), trustability, τ, was used for success, while 1−τ was for failure; *G* represents the gain, and *L* denotes the loss. Then, the cost of resources was deducted, representing the sum of the cost of all nodes for the cloud scenario.

(10) $$U_{net}=\tau G-\left(1-\tau \right)L-\sum C_{node_{i}}$$

In Section 3, trustability results are presented for the scenarios wherein such systems with different configurations experience different incidents, thus causing node and system-level trustability declines.

## 3. Results and Discussion

This section presents the results for two types of attacks on systems. First, the effects of external attacks on systems already deployed in the field are presented as well as the possible actions to take afterward. For instance, a sensor could lose its trustability by providing either inadequate or unreliable data. Furthermore, results and potential cautions are discussed after a decline in the trustworthiness of a service running on the cloud due to internal anomalies. In such a scenario, a service runs on multiple nodes deployed on the cloud, and a subset of the nodes loses trustability due to various internal factors such as high usage of processing power, memory, or bandwidth or a compromised task running on a node.

### 3.1. External Attacks

**Figure 3:**
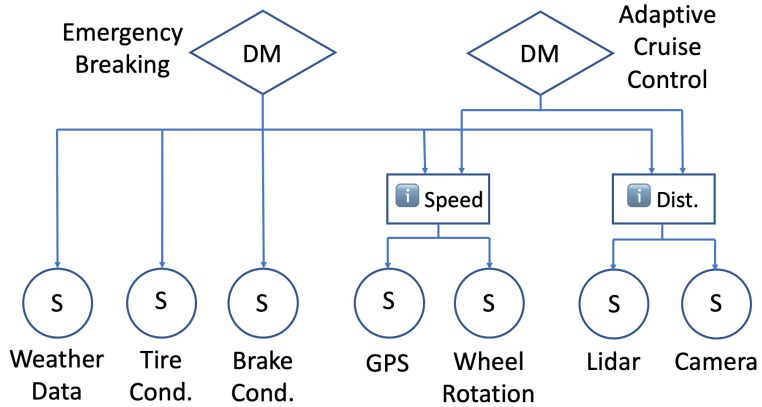
Sample diagram of an active safety system in a vehicle.

This section explores the external attacks and related factors on the sensors and subsystems of a system running in the field. In addition, the trust management system is demonstrated by capturing such conditions and facilitating decision making. In Figure 3, a sample diagram of an active safety system is shown in a vehicle that has multiple sensors and two decision-making mechanisms. In real systems, higher levels of hierarchies among decision makers and sensors are predicted [13].

For example, a vehicle with an active safety system is expected to make decisions based on the information it receives from the sensors. However, such information may not always be reliable due to compromised sensors or altered sensor data. In such cases, the decision-making mechanism should be able to take the necessary actions for the safety and reliability of the decision. For instance, a dead battery in a tire pressure sensor could cause an incorrect tire condition measurement that would then affect the braking decision. Similarly, malware in a camera system that measures the distance to the vehicles in front could cause an erroneous distance measurement, which is crucial for emergency braking and adaptive cruise control.

**Figure 4:**
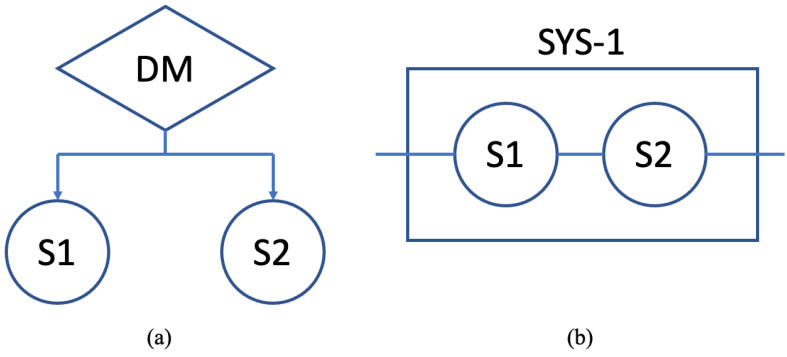
Sample system (System-1) with one decision maker (DM) and two sensors, S1 and S2. (**a**) System diagram as DM relies on S1 and S2. (**b**) Logic representation where the sensors are connected in series.

The systems of sensors and decision-making mechanisms could quickly get complicated with multiple layers and hierarchies. We illustrate our trustworthy approach by using a simple system that can be considered a subsystem of the entire mechanism. In the first system, System-1, one decision-making element (DM) relies on two sensors, S1 and S2. It receives information from these sensors and makes a decision. This system can also be represented as a logical system, where the sensors are connected in series. Figure 4 shows the sample system and its logic representation.

**Figure 5:**
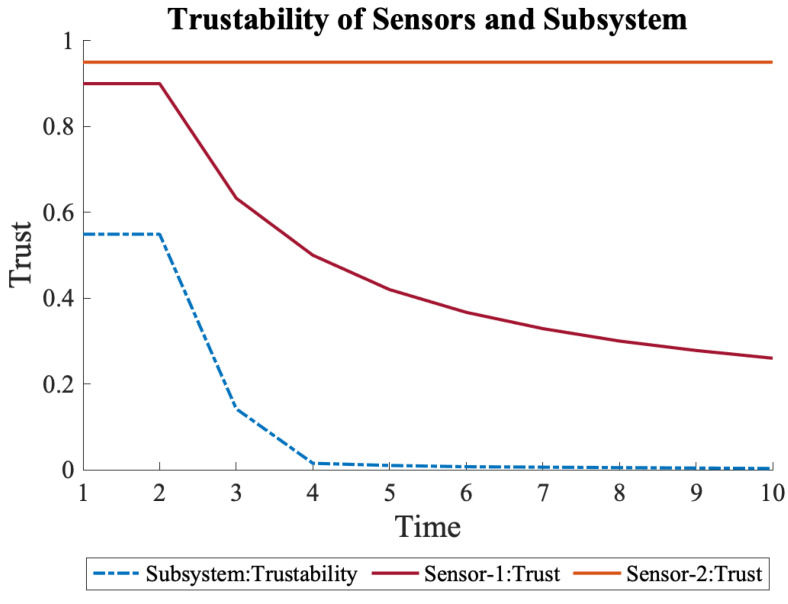
Trustability of System-1 and the sensors. The subsystem is susceptible to any trust fluctuation due to serially connected sensors.

We explored a scenario wherein the trust of the first sensor, S1, declines. In the beginning, both sensors were assumed to have high trust, 0.90 and 0.95, respectively, because of consecutive measurements with the same values, as shown in Equation (11). However, due to a hypothetical external attack, the value of S1 became 0.1, which caused trust to decline, as shown in Figure 5. This also caused a decline in the trustability of the system, which was calculated using Equation (7).

(11) $$\begin{array}{ccc} S1 & = & \left\{0.9,0.9,0.1,0.1,0.1,0.1,0.1,0.1,0.1,0.1\right\}\\ S2 & = & \left\{0.95,0.95,0.95,0.95,0.95,0.95,0.95,0.95,0.95,0.95\right\} \end{array}$$

**Figure 6:**
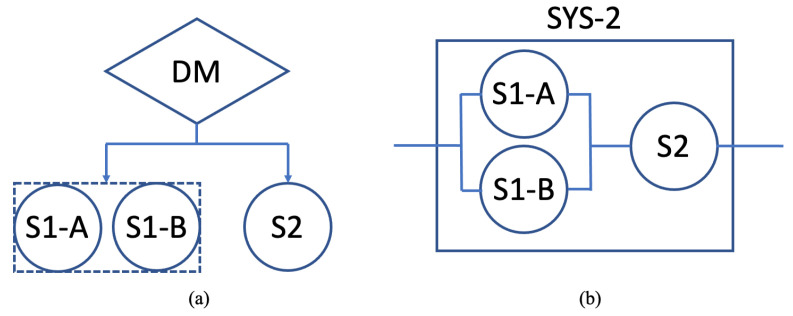
Sample system (System-2) with one decision maker (DM) and three sensors, S1-A, S1-B, and S2. (**a**) System diagram as DM relies on three sensors, where S1-A and S1-B are used for the same information. (**b**) Logic representation, where S1-A and S1-B are connected in parallel and are connected to S2 in series.

One option could be to include another sensor for the same task in order to overcome the adverse effects of losing the ability to make trustworthy decisions as a result of one sensor failure. The system was updated such that the DM could rely on two sensors, S1-A and S1-B, for the information that previously required its reliance only on S1. Figure 6 shows the diagram of System-2 and its logic representation, where S1-A and S1-B are connected in parallel.

**Figure 7:**
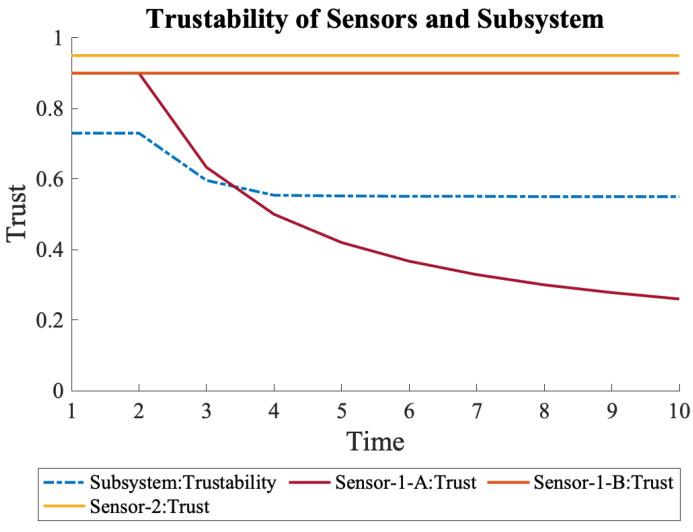
Trustability of System-2 and the sensors. The overall trustability of System-2 is more resistant to the trust surges of the sensors that are connected in parallel.

When the previous scenario occurred and S1-A’s trust declined, the overall trustability of System-2 did not decrease as it did in System-1. The initial trustability of System-2 was also higher than that of System-1 due to the fact that it had the additional sensor, S1-B, in the system from the beginning. Figure 7 shows the change in the overall trustability of System-2 and the trust of the sensors over time.

**Figure 8:**
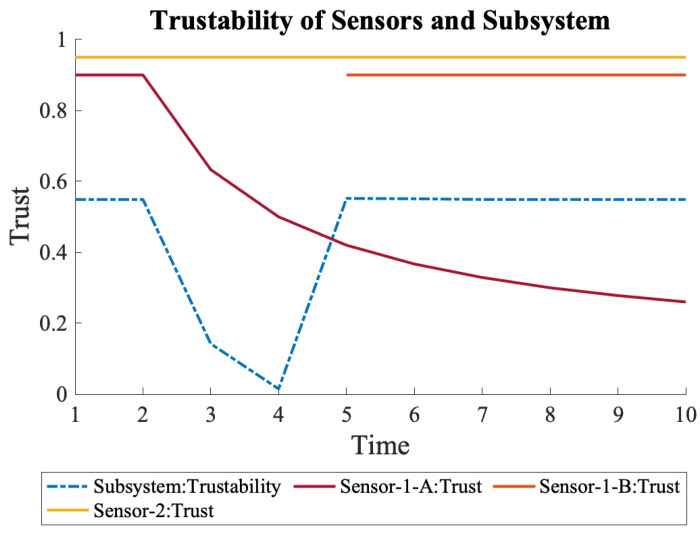
The overall trustability of System-3 and the sensors. S1-B is only activated after distrust of S1-A decreases the system’s trustability below a threshold. Activation takes time.

Another scenario is to have the additional sensor, S1-B, in the system, but to have it activated only when needed. When the trustability of the system is below a specific threshold, another sensor is activated to bring the overall trustability back to an acceptable level, as shown in Figure 8. A grace period for sensor activation was added to reflect a more realistic scenario. This scenario could happen when a sensor has a short lifetime and is only activated when the other sensor does not satisfy the requirements anymore. One drawback of such an approach is the lower initial trustability as compared to when all sensors activated, which can also be observed when Figure 5 and Figure 7 are compared.

### 3.2. Internal Incidents

This section explores the incidents affecting some cloud nodes that run a service. The trust management framework captured the decline in the trustability of the service and nodes and assisted in taking timely actions to keep the service reliable. Although today’s cloud infrastructures can be very complicated and highly hierarchical, the scenarios were built on a sample cloud that had three nodes, with each running a different task.

Internal incident scenarios on cloud services differ from the system scenarios with sensors explained in Section 3.1. While the number of sensors should be decided before the device’s production, cloud scenarios have more flexibility. A task can be migrated to another node, or alternative nodes can be launched. This also brings dynamic cost optimization into the picture since the nodes can be dynamically added and removed.

**Figure 9:**
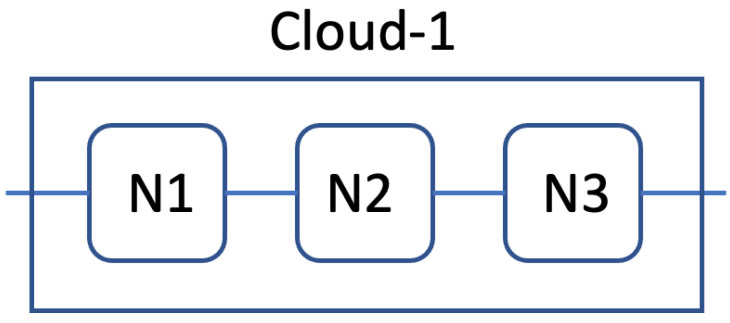
Sample cloud structure consisting of three nodes, each running a different task.

The sample cloud architecture consisted of three nodes connected in series, which means that the service needed three tasks deployed on different nodes. Since the reliability of the service depends on all three nodes, those nodes can be considered connected in series, as shown in Figure 9.

**Figure 10:**
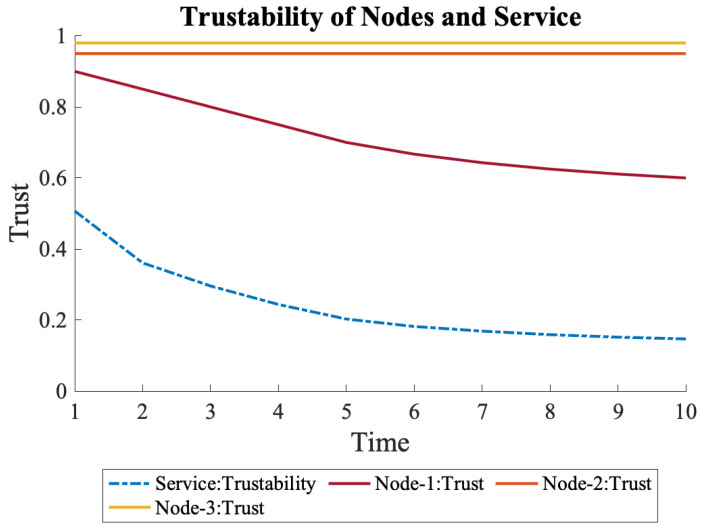
Trustability of a service running on three nodes on the cloud. A decline in the trust of N1 causes a decline in overall trustability.

Four different scenarios were explored, as introduced in Equation (5), each of which caused other declines in the trustability of the service. For each scenario, only the first node, N1, was affected. In the first scenario, N1’s trust measurements declined slowly until time 5 and then stayed the same, as shown in Equation (12). This caused a slow decline in the trust of N1 until time 5, which subsequently started settling gradually. However, since the nodes were connected in series, even a slight decrease in trust for one of the nodes caused a considerable decline in the trustability of the service deployed on the cloud. Figure 10 shows the change in the trustability of the service and the nodes.

(12) $$\begin{array}{ccc} N1 & = & \left\{0.9,0.8,0.7,0.6,0.5,0.5,0.5,0.5,0.5,0.5\right\} \end{array}$$

**Figure 11:**
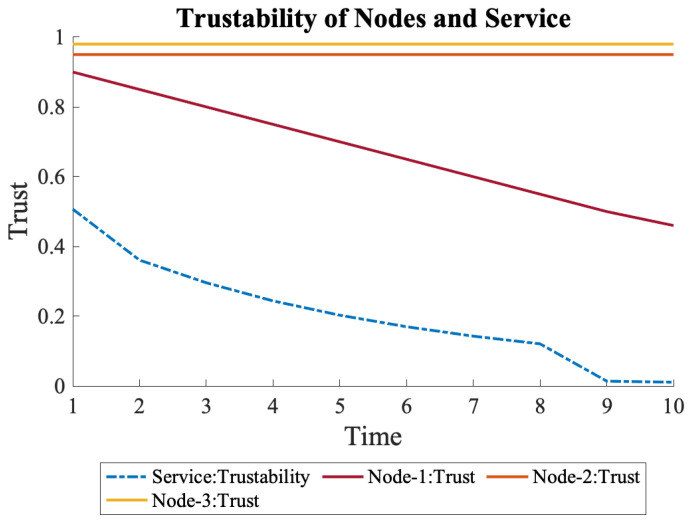
When trust of N1 continuously declines, its effects on the overall trustability is more severe.

When the decline in trust of N1 continued, as shown in Equation (13), it placed more significant stress on the overall trustability of the service. It even decreased the overall trustability to almost zero, as shown in Figure 11, which is a sign that the system is not trustworthy anymore due to the low trust of N1.

(13) $$\begin{array}{ccc} N1 & = & \left\{0.9,0.8,0.7,0.6,0.5,0.4,0.3,0.2,0.1,0.1\right\} \end{array}$$

**Figure 12:**
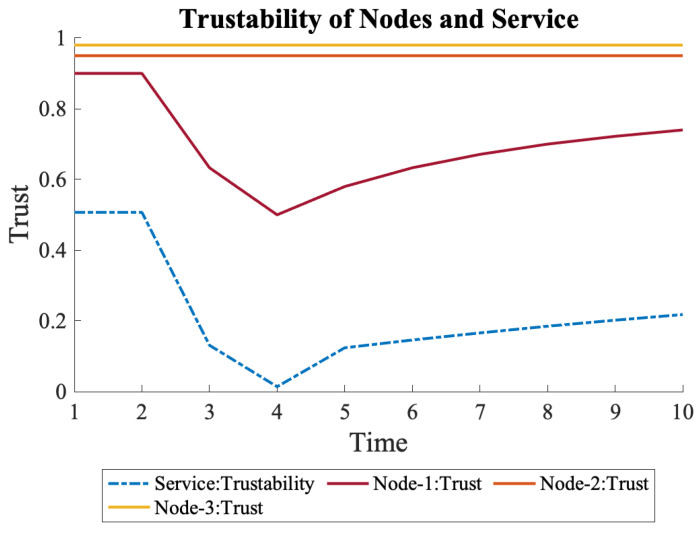
If the trust declines rapidly for a short period of time, overall trustability also declines but does not recover quickly.

Furthermore, a sudden decline in the trust measurements of N1 was explored, which returns to normal after some time, as shown in Equation (14). For example, in this case, while the regular trust measurements were at 0.9, it dropped to 0.1 at times 3 and 4 due to an internal anomaly, such as high processing power, memory, or bandwidth usage. As shown in Figure 12, the trust of N1 gradually recovered; however, the overall trustability dropped close to zero at time 4 and recovered slowly due to historic trust measurements.

(14) $$\begin{array}{ccc} N1 & = & \left\{0.9,0.1,0.1,0.1,0.9,0.9,0.9,0.9,0.9,0.9\right\} \end{array}$$

**Figure 13:**
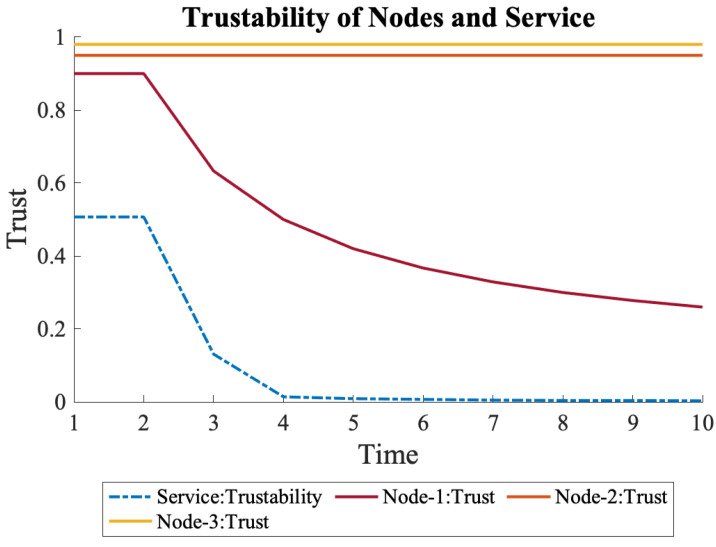
A sudden drop in trust measurements causes a continuous decline in the trust of N1. This reflects a more dramatic decline in the overall trustability of the service.

In the final scenario, the sudden decline in the trust of N1 was set to be permanent. When the trust of N1 did not recover, as shown in Equation (15), the trustability metric did not recover either, as shown in Figure 13. As in the previous scenario, it declined close to zero, indicating the service’s low trustability.

(15) $$\begin{array}{ccc} N1 & = & \left\{0.9,0.1,0.1,0.1,0.1,0.1,0.1,0.1,0.1,0.1\right\} \end{array}$$

In response to the decline in the trustability of the service, some measures can be taken. One example is to use alternative nodes for each task. It provides the service to utilize both nodes for the specific task. However, this option comes with a cost since the service provider would be charged for each node operated. As we mentioned previously, a more sophisticated approach would be to use the nodes when needed.

**Figure 14:**
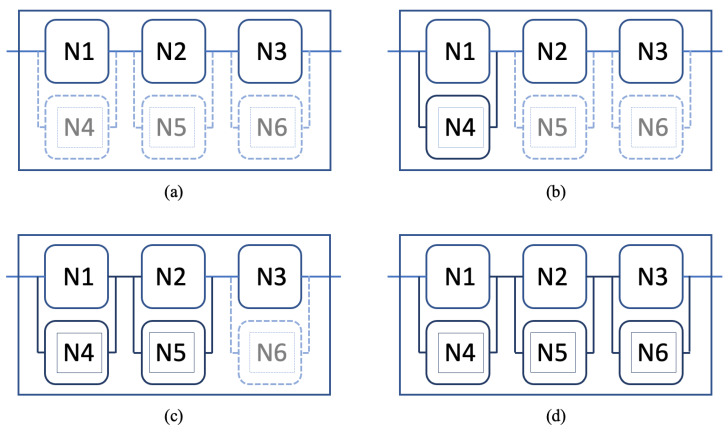
Cloud structure updated with additional nodes for each task. (**a**) The original Cloud-2 with N1, N2, and N3, including the options to add N4, N5, and N6. (**b**) N4 is added after N1’s trust declines. (**c**) N5 is added after N2’s trust declines. (**d**) N6 is added after N3’s trust declines.

As shown in Figure 14, Cloud-2 can add a node for each task and remove them on demand. A new scenario was explored where the trust of each primary node, N1, N2, and N3, declined gradually, starting at different times. After a grace period of 1, a new node was launched for each task to increase the overall trustability.

Once N1’s trust started to decline at time 2, the overall trustability of the service also decreased. After a grace period, N4 was activated at time 4, which increased the trustability of the service again. The new trustability was more than the initial trustability since N1 was still active despite the lower trust. At the same time, N2’s trust started to decrease, which was compensated by launching N5 at time 6. Finally, N3’s trust started to decline, and N6 was launched.

**Figure 15:**
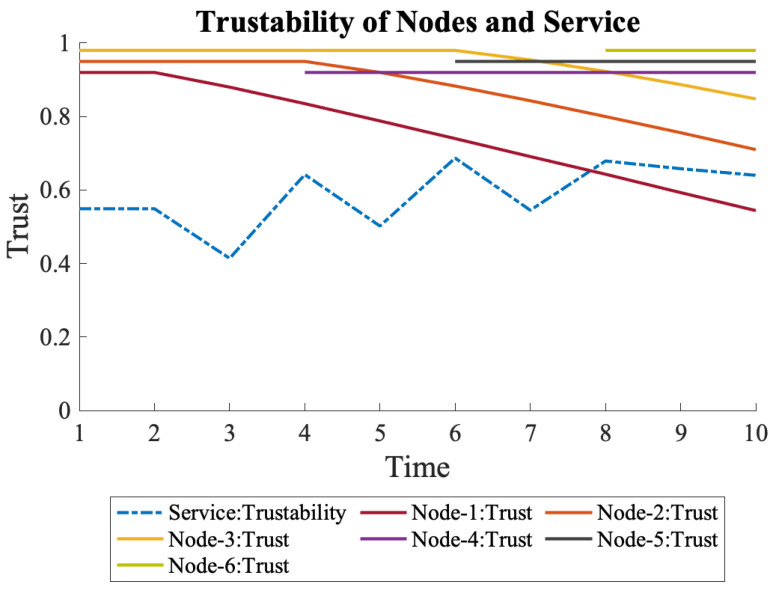
The entire trustability of the system increases each time a new node is launched and connected in parallel to the initial nodes.

As seen in Figure 15, the overall trustability surpassed the initial value since the old nodes were still active and had nonzero trust values. Another conclusion is that the system became more resilient to individual trust declines once the nodes started having alternatives, such as having additional nodes that were connected in parallel.

Since the continuous decline in trust of the initial nodes keeps decreasing the overall trustability, deactivating those nodes can be considered. Deactivating a parallel connected node with a nonzero trust would also cause a decrease in trustability; however, it is worth exploring the degree of decline resulting from the cost of the nodes.

**Figure 16:**
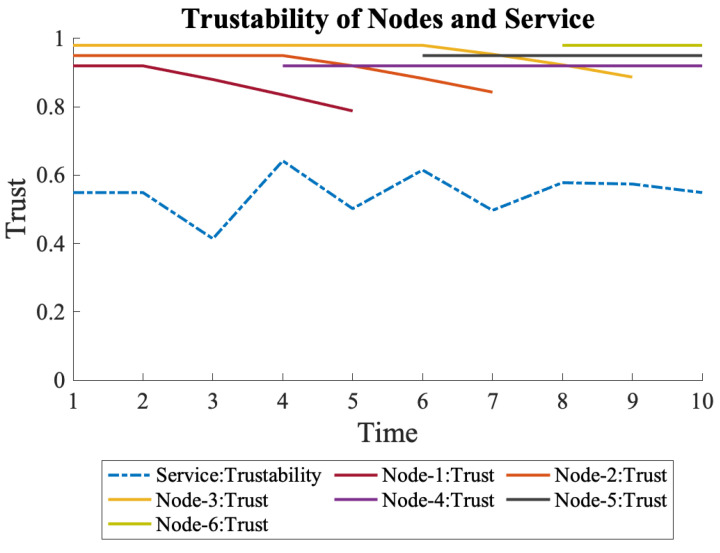
Deactivating a node that is losing trust is one way to keep costs low with the least sacrifice in the overall trustability of the service.

Figure 16 represents each node’s overall trustability and trust when a node is deactivated after a decline in trust. Compared to Figure 15, where the initial nodes are not deactivated, the overall trustability is lower. However, the advantage appears when the cost of the service is considered.

**Figure 17:**
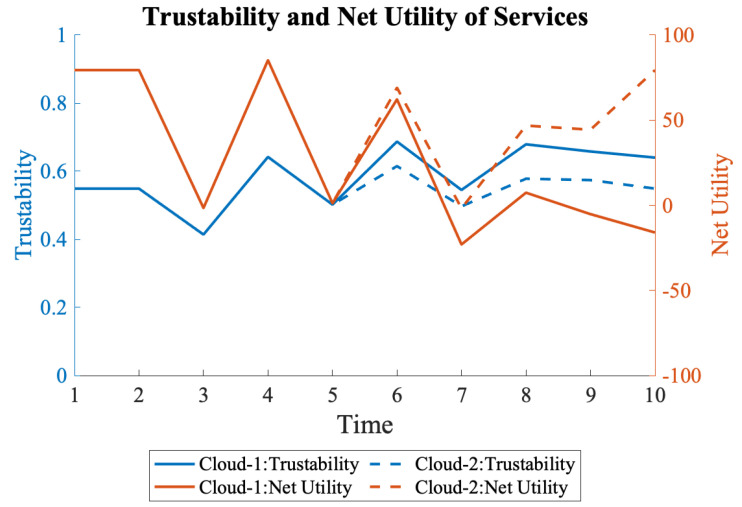
Trustability and net utility of Cloud-1 and Cloud-2. Cloud-1 has higher trustability but lower net utility due to more active nodes.

The net utility of the system was calculated using Equation (10). The gain of a running service was assumed to be 500 units, the loss of a down system was considered to be 100 units, and the cost of an active node was assumed to be 50 units. Figure 17 shows the trustability of services running on Cloud-1 and Cloud-2 and their net utilities. Cloud-1 has higher trustability starting at time 4, when the nodes’ deactivation started in Cloud-2. The final trustability of Cloud-1 and Cloud-2 are 0.64 and 0.55, respectively, showing a 14% decrease. However, looking at the net utility, which also considers trustability, Cloud-1 has a negative net utility of -16, whereas Cloud-2 has 79.

The result of the net utility function is highly dependent on the choice of gain, loss, and node cost values. In this paper, we identified the importance of employing a trust management framework to actively observe the trustability of the service and each node in order to take the necessary actions that would proactively keep the service alive.

## 4. Conclusions

This paper explored the external and internal attacks and incidents occurring in systems with connected sensors and a service deployed on multiple nodes on a cloud. First, two systems consisting of a decision maker and sensors were compared, where the second system had an extra sensor for redundancy. The proposed trust management framework successfully captured the trustability of the sensors and the entire system in both scenarios. An alternative scenario was explored wherein the extra sensor could be activated when necessary, and the results were compared with the prior scenarios.

Furthermore, a sample cloud was simulated with three nodes, where the trust of a node decreased due to some internal incident. The trustability metric captured the overall trustability decline for different incident types. Then, the cloud was updated to add and remove nodes on demand. Each task was supported by an extra node when the individual trust declined in order to keep the overall trustability of the service high. Finally, the cloud systems with and without node deactivation were compared in terms of their trustability measurements and the net utility of the comprehensive service.

The results showed that the utilization of our trust management framework helps in deciding how to mitigate severe consequences of external attacks on sensors or internal incidents on a cloud while considering the net utility of the system. One of the difficulties in this work could be the measurement of the trust of the system parts, such as a sensor or a node, which requires field knowledge for different scenarios; however, it is out of the scope of this paper.

This work can be further extended by considering more realistic and complex scenarios, which include multi-level hierarchies of sensors or nodes. Moreover, a decision maker may rely on other decision makers in addition to sensors. Similarly, a service may depend on other services in addition to its tasks. These scenarios may require the trustability metric formula to be adjusted to specific situations. Future work could also include the cost of trustability measurement operations in the utility function since the historic computation of trust could surge as the number of individual measurements and components increases. Consequently, shifting the defense mechanism from classical, perimeter-based approaches to system resilience is considered to be a long-term objective.

## Data Availability

Not applicable.

## Abbreviations

The following abbreviations are used in this manuscript:

5G	Fifth Generation
6G	Sixth Generation
CISA	Cybersecurity and Infrastructure Security Agency
IoT	Internet of Things
MEC	Multi-access Edge Computing
AI	Artificial Intelligence
DM	Decision Maker
S	Sensor
N	Node
UAV	Unmanned Aerial Vehicle
US	United States
USDA	United States Department of Agriculture
NIFA	National Institute of Food and Agriculture
NSF	National Science Foundation
